# 

**Published:** 1879-12

**Authors:** 


### INDEX TO VOLUME XXXIX.

A.

PAGE.

Abdominal cavity, drainage of. 92
Abcess, frontal, recurrent .. 482
Academy of medicine, dedication of the new library hall of.... 559
Address, abstract of, Moses Gunn M.D.... 50
Address, abstract of, Dr. E. S. Lewis.... 53
Address, abstract of, Dr. H. Knapp.... —
Address, abstract of, Dr. J. S. Billings .... 45
Address, abstract of, Dr. T. F. Rochester .... 43
Address, abstract of, Dr. Theophilus Parvin.... 40
Address, abstract of, C. T. Parkes M D.... 470
Alienist and Neurologist, notice of .... 445
Alcoholism, E. C. Helm, M.D.. 623
American Medical Association, reports of.... 39
American Public Health Association ....334, 519
American Academy of Medicine.... 221
American dermatology, rise of. 385
American dermatological association, proceedings of, at third annual meeting, reported by J. N. Hyde, M.D. .... 385
Anæsthetics .... 551
Andrews, Edmund, MD., the corn-doctor's progress.... 24
Amyl nitrate, as a cardiac stimulant .... 104
Announcements for months, 112, 224, 336, 448, 560, 672
Announcement of tri-State Medical Society of Indiana, Illinois and Kentucky .... 508
Appointments ....290, 384

PAGE.

Arteries, radial, hereditary variation in, by J. Schneck, M.D. 475
Aspiration of knee-joint, by Dr. H. O. Marcey .... 67
Autopsy Society, Mutual, of Paris, by H. M. Lyman, M.D. 373
Autopsy.... 466

B.

Bartlett J., M.D., sundry facts, opinions and suggestions derived from the observation of forty cases of tapeworm.... 244
Beef and ice cream, by J. I. Tucker, M.D.... 38
Billings, J. S., M.D., abstract of address.... 45
Boeckel, Jules, M.D., catgut drainage .... 632
Book Notices and Reviews:
Bilroth's general surgical pathology and therapeutics in fifty-one lectures.... 324
Fox's photographic illustration of skin diseases.... 209
Farquharson's Guide to therapeutics and materia medica 208
Green's introduction to pathology and morbid anatomy. 325
Gegenbauer's elements of comparative anatomy.... 325
Landoldt's manual of examination of the eyes.... 206
Neubauer & Vogel's guide to the qualitative and quantitative analysis of the urine... 211
Leffingwell's reading book of English classics.... 210
Ranney's practical treatise on surgical diagnosis.... 323
Ellis' demonstration of anatomy; being a guide to the knowledge of the human body by dissection.... 323

PAGE.
Bucknill's habitual drunkenness and insane drunkards. 321
Report of the Department of Health of City of Chicago for the year 1878........ 309
Bartholow's spermatorrhœa; its causes, symptoms, results and treatment ............. 441
Philosophical problems in medicine.................. 440
Therapeutics and materia medica, by H. C. Wood.... 637
First step in chemical principles, Henry Leffmanberg.. 638
Conspectus of organic materia medica, L. E. Sayre....... 637
Hygiene and public health, A. H. Buck................ 639
Chemistry—general, medical and pharmaceutical, Attfield 638
Guide to the examination of urine, H. B. Hoffman..... 638
Ostler's pathological report of the Montreal General Hospital for year ending May 1, 1877, notice of ... ..... 326
Bowel affections in young children, etiology of, by N. S. Davis, M.D ................ 364
Brain, congestion of, bleeding.. 490
Brower, D. R., M.D., traumatic insanity.................... 609
Bushnell, K. C., the process of inflammation ........... 155
Bullous eruption induced by the injection of iodide of potassium, contribution to the study of........ ........... 389
Byford, W. H., M.D..... 202, 335
Byford, W. H., M.D., ergot in the treatment of fibroid tumors of the uterus................ 337

C.

Capillary fat embolism of lungs and brain, Chr. Fenger, M.D.. 587
Cancer of stomach, ulcerated, by J. R. McCullough, M.D.... 462
Cancer of stomach, differentiation of from dyspepsia by abdominal temperature... .... 267
Cardiac disorder, acute inflammatory, a conspectus of three different forms of, by Roswell Park, M.D.................. 349
California's last, best gifts..... 110
Calculus detained in urethra... 90
Caffein citrate of as diuretic in cardiac dropsy............. 91

PAGE.
Changes in editorial staff...... 105
Chicago Medical Society .. ... 631
Chorea, treatment of........... 548
Chronic Rheumatic carditis, clinical lecture, by N. S. Davis, M.D ................... 225
Church, N. H., M.D., case of complete Procidentia Uteri cured by operation................ 273
Chloral hydrate enemata preparatory to ovariotomy, by C. H Hughes, M.D.............. 382
Cholagogues .................. 333
Chancre of lip, probably acquired through cigars....... 391
Chicago Medical Society, report of......................... 88
Chloroform, administration (discussion)................... 88
Chloroform poisoning, by P. O'Connel, M.D........... .. 379
Chloroform, administration of, by Wm. Meacher, M.D....... 240
Cleft palate .......... ...... ... 92
Clavicle treatment of fracture of, discussion ............ 6
Correspondence, Dudley E. C. M.D., to Barnes R., M.D. . .... 101
Corn-doctors' progress, by Edmund Andrews, A.M. M.D.... 24
College fees ... ............... 205
Correction of Dr. Caldwell, by Dr. E. T. Tappey............ 185
Correction of Dr. Caldwell, Dr. Westphal .................. 184
Correction....... ........220, 285
Conspectus of three different forms of acute inflammatory cardiac disorder, by R. Park, M.D ........................ 349
Corporal punishment, by P. P. Marsh, M.D................. 295
Convulsions in children, treatment of ... ................ 550
Colon, malignant stricture of the hepatic flexure of, by J. E. Goodhart, M.D ........... 523
Colleges, more medical ....... 522
Concours, Rush medical college........ ................ 669
Cranioclasm, spontaneous ..... 167
Cranii basio, fracture of........ 257
Croup or diphtheria (discussion)...................... 92
Cuprum ammoniatum in neuralgia of the fifth........... 104

D.

Davis N. S. M.D., origin and spread of yellow fever....... 574

PAGE.
Davis N. S., M.D., chronic rheumatic carditis; senecio aureus as a remedy in the rheumatic diathesis .... 225
Etiology of bowel affections in young children .... 364
Delegates to foreign medical societies .... 56
Death registration for census .. 10
Dermatological society, announcement of .... 196
Dermatitis Venenati, discussion of .... 422
Dental hints for country practitioners, G. M. Newkirk, M.D. 615
Diseases, infectious, the British Medical Association on the registration of .... 427
Diarrhœa infantile, discussion of .... 425
Diaphragm and stomach, rupture of, clin. report .... 268
Dispensaries, Boston letter .... 509
Dilator, new uterine, by J. O. Hobbs, M.D .... 468
Digestion, artificial, MM. Vulpian and Mourrut .... 666
Doremus, R. Ogden, epidemics from a chemical standpoint .. 538
Drainage of abdominal cavity .. 92
Dropsy of the amnion, three cases, by F. C. Robinson, M.D. 32
Drainage, catgut, Jules Boeckel 632

E.

Ear diseases, clinical lecture by S. J. Jones, M.D .... 449
Earle, C. W., M.D., tubercular meningitis .... 11
Eczema and ulcers of leg by an elastic tubular bandage .... 394
Editorials .... 515, 299, 105
Educational progress .... 335
Elgin Insane Hospital appointment .... 38
Embolism .... 284
Embolism, capillary fat embolism of lungs and brain, Dr. Chr. Fenger .... 387
Epitaph .... 290
Epidemics from a chemical standpoint, by R. Ogden Doremus, M.D .... 538
Epley, F. W., M.D., a case of transfusion .... 234
Ergot in the treatment of fibroid tumors of the uterus, by W. H. Byford, A.M., M.D .... 337
Etiology (skin diseases) .... 416

PAGE.
Everett, M.D., J. T., anomalous complication of uterine fibroids .... 598
Explanation .... 308

F.

Fenger, Dr. Chr., fat embolism of lungs and brain .... 587
Fibro-myoma of uterus, pedunculated, by O. Stroinski, M.D. 375
Fibroid tumors of uterus, Ergot in .... 37
Fitch W. H.,
Tinea Kerion .... 478
Pustular eczema of scalp .... 480
Fibroids, uterine complications of, Dr. J. T. Everett .... 398
Forceps in first stage of labor, discussion .... 92
Fox Tilbury, death of .... 108
Fœtus, alterations of the deadborn .... 552
Fracture, a rare, by E. C. Huse, M.D .... 175
Fungoid neoplasm, inflammatory, supplement to a case of. 413
Fungus hæmatodes and melanotic carcinoma .... 486

G.

Giralt, Dr., jaborandi in yellow fever .... 667
Goodhart, J. E., malignant stricture of the hepatic flexure of the colon .... 523
Gunn, M.D., Moses, address, abstract of .... 50
Guillotin *vs.* carbonic oxide .... 473

H.

Hard times for professional men in England .... 455
Hart, J. N., M. D., practical hygiene, etc .... 135
Hard, Chester, M.D., hypertrophy of the heart and effusion within the pericardium, case of .... 36
Hæmorrhoidal tumors by carbolic acid, discussion .... 71
Health Department in Hyde Park, by N. H. D. Lewis, M D. 201
Hernia reduced by elastic bandage, by R. Tilley, M.D .... 279
Hermaphordism false, Drs. Arigo and Fiorani .... 664
Helm, M.D., E. C., Alcoholism .... 628

PAGE.
Heitzman C, microscopical studies on inflammation of the skin.................... 396
Hindoo medical science....... 223
Hip-joint disease, C. T. Parkes, M.D ......................... 199
Hobbs J. O., M.D., a new uterine dilator........................ 468
Hotz, F. C., M.D., aneurism of eyelid, chloroform asphyxia.. 88
Hospital reports, Cook county.
Periodical mania......... 28
Cancer of gall bladder and multiple abcess of liver.. 29
Stricture of trachea....... 31
Hotz F. C., M.D., a singular plug in external meatus....... ... 383
Hughes C. H., M.D., chloral and hydrate enemata preparatory to ovariotomy............ ... 382
Huse E. C., M.D,, fracture, rare case of....................... 175
Hyde, J. N., M.D............... 335
Hypertrophy of heart and effusion within the pericardium, case of, by Chester Hard, M.D. 37
Hydrophobia, case of, with autopsy, by H. M. Lyman, M.D. 34
Hydrophobia, case of, with brief general review of disease, by N. Senn, M.D............... 145
Hygiene, practical, as it relates to a proper water supply, by J. N. DeHart, M.D........... 135
Hyde, J. N., M.D., Proceedings of the American Dermatological Association at third annual meeting, report of...... 385
Hypodermic injection of morphia, by H. H. Kane, M.D.... 378
Hyde, James Nevins, M.D., clinical lecture on tubercular leprosy ......................... 561

I.

Illinois State Medical Society.. 203
Inflammation, process of, by K. C. Bushnell, M.D........ .... 155
Inhaler, new ether............ 73
Information, useful............ 436
Intestinal obstruction.... ..... 522
Indications for surgical operations, M. Verneuil........... 667
Ingals, E. F. M.D, laryngeal tumors and tubercular laryngitis ....................... 1
A bone in the larynx for eight months .................. 468
Iritis tubercular................ 328

PAGE.
Iron, dialysed, constitution and properties of. ............... 495

J.

Jaborandi in yellow fever, Dr. Giralt ...................... 667
Jackson A. R., M.D., laceration of cervix .................. 113
James' Powder, true composition and therapeutical uses of. by P. O'Connell, M.D........ 160
Jones, S. J., M.D., diseases of the ear.... .................... 449
Kane, H. H., M.D., hypodermic injection of morphia... .... 378
Keitz, Gustave, remarkable case of opium poisoning......... 276
Kentucky, Louisville, medical colleges ..................... 327
Knapp, I. C., M.D., abstract of address ..................... 57

L.

Laceration of cervix uteri, by A. Reeves Jackson, M.D...... 113
Laceration of cervix uteri..... 308
Laceration of cervix ut'ri (discussion)..................... 288
Larynx, bone in, for eight months, by E. F. Ingals, M.D., 474
Laryngeal tumors and tubercular laryngitis, by E. F. Ingals, M.D .................... 1
Lead poisoning from the use of baby carriages................ 187
Lewis, E. S., M.D., abstract of address ..................... 53
Lewis, W. H. D., Health Dept. in Hyde Park............... 207
Lectures on physical and functional examination of the eye, 1
announcement of............ 431
Lectures on skin diseases, announcement of............... 431
Letter, New York.............. 6[illegible]3
Letter, Boston ................ 195
Legs, loss of both............. 384
Leprosy tubercular, lecture on, by J. N. Hyde, M.D.......... 561
List of medical officers of the U. S. Marine Hospital...... 109
Lyman, H. M., hydrophobia, case of, with autopsy........ 34
Lyman, H. M., M.D., the Mutual Autopsy Society of Paris.. 373

M.

Mastoid abcess, by J. A. Goldsberry, M.D .. .............. 269

PAGE.
Marsh, B. P., M.D., corporal punishment.... 295
Martin's bandage, by H. A. Martin, M.D..... 188
Martin, H. A., M.D., Martin's bandage.... 188
McCullough, J. B., M.D., ulcerated cancer of stomach...... 463
Medical education, resolutions on..... 81
Meacher, Wm., the administration of chloroform.......... 240
Meatus external, a singular plug in, by F. C. Hotz, M.D...... 383
Medical classes.............. 439
Medical colleges ............ 308
Medical societies............. 299
Merriman, H. P., M.D., perimetric inflammation........... 456
Meningitis, tubercular...... 11
Metric system.............. 53
Michigan State Board of Health 286
Missing link.... .......... 515
Michigan State Board of Health, report of.................. 501
Molluscum verrucosum, a variety of, presenting certain unusual features............... 414

N.

Nævi tattooing.. ............ 409
National and State Boards of Health.................. 303
National co-operation with State and local Boards............ 303
New instruments, by E. B. Turnipseed, M.D............... 68
Neubauer & Vogel's guide to the qualitative and quantitative examination of the urine 211
New York letter.............. 291
New York letter.............. 633
Newkirk, G., M.D., dental hints. 615
Nominations of Illinois State Medical Society.......... ... 80
Nominations of American Medical Associations............ 47

O.

Obituary, Theodore W. Stull, M.D., by Abner Hard, M.D.... 219
Obituary, Hatch Ira, Baker, S. F.......................... 557
Obituary of D. H. Speckler.... 670
Obituary of A. S. Brown, M.D.. 670
O'Connell, P., M.D., composition and therapeutical uses of true James' powder.............. 160

PAGE.
O'Connel, P., M.D., was it poisoning by chloroform?......... 379
Officers, election of, of American Dermatological Association........................ 405
Opium poisoning, case of, by Gustav Keitz, M.D........... 277
Origin of plants.............. 663
Ovaries, extirpation of both in hystero epilepsy............. 444
Ovariotomy, results of......... 548

P.

Paralysis following labor, by A. E. Hall, M.D................ 476
Park, Roswell, M.D., conspectus of three different forms of acute inflammatory disorder.. 349
Parkes, C. T., M.D., hip-joint disease..................... 179
Parvin, Theopilus, M.D., abstract of address................. 40
Perineal operations, state of bowels after................. 62
Peritonitis .................. 438
Permetric inflammation, discussion of..................... 496
Perimetric inflammations, by H. P. Merriman, M.D........ 456
Perimetric dimension system, C. H. Thomas, M.D ......... 658
Phosphaturia in its relations to surgical operations.......... 205
Phlegmasia alba dolens, clinical reports.................... 167
Physicians vacations.......... 306
Pilocarpine chlorhydrate, in the production of permature labor...................... 166
Progress, gratifying........... 508
Preventive medicine... ....... 432
Pustular eczema of scalp, by W. H. Fitch, M.D.... ......... 480

R.

Reports, medical societies in London..................... 92
Reports, Chicago Med. Soc.... 88
Report of committee on nominations, Amer. Med. Ass'n ..
Reports, Chicago Gyn. Soc..... 178
Robinson, F. C., M.D., three cases of dropsy of the amnion..... 32
Rochester, T. F., M.D., abstract of address................. 43
Rickety pelvis, peculiarity of, 212
Rubber bandage ............ 429

PAGE.

S.

Salisbury, J. H., M.D.:
Periodical mania..... ..... 28
Cancer of bladder and multiple abscess of liver....... 29
Stricture of trachea.......... 31
Salisbury, J. H., M.D., a case of rupture of the diaphragm and stomach.................... 208
Sarcoma, malignant........... 484
School hygiene.............. 86
Scrofula and scarcity of food in London.................... 446
Schneck, J., M.D., hereditary variation in the radial arteries... 475
Septicæmia, Hervieux and Pasteur on ................... 215
Septicæmia, clinical report ... 167
Senn, N., M.D., a case of hydrophobia with brief general review of disease............... 145
Sewerage, defective of cities and towns, hospitals and asylums, by J. N. DeHart, M.D........ 135
Senecio aureus as a remedy in the rheumatic diathesis, by N. S. Davis, M.D................ 225
Skin, inflammation of, microscopical studies, Heitzman, C. M.D......................... 396
Shoulder-joint, excision of, for necrosis........ ........... 487
Smith, W. H., M.D., poisoning by strychnine—prompt recovery after administration of potassium bromide.... ....... 280
Society reports, American Medical Association............. 39
Soap for physician's office..... 426
Spine, carious fragments coughed up .... ................. 91
Speckler, D. H., M.D., obituary of......................... 670
Spina bifida, Dr. L. H. Montgomery......................... 631
Stroinski, O., M.D., extirpation of a pedunculated fibro-myoma of uterus; recidive showing all the characteristics of sarcoma..................... 375
Strychnine poisoning, case of by W. H Smith, M.D........ 280
Suicides ..................... 522
Suppositories gelatine.. ...... 636
Surgical operations, indications for, M. Verneuil............ 667
Syphilis in relation to marriage. 216
Syphilis, bone hereditary...... 332
Syphilis acquired, infantile.... 331

PAGE.

Syphilis, hereditary transmission of.................. 332
Syphilis, infection of a nurse by her nursling............ ... 330
Syphilis by vaccination with human virus................ 330
Syphilis, the nature of......... 419

T.

Tapeworm, forty cases of, by J. Bartlett, M.D................ 244
Tape-worm in a fish........... 212
Tappey, E. F., M.D., correction of Dr. Caldwell............. 184
Tetanus traumatic, two cases of, G. H. Salisbury, M.D......... 627
Thermometer, a urine, for gynæcological practice........... 439
Thomas, C. H., M.D., perimetric dimension system.......... 658
Tilley, R., M.D., hernia reduced by elastic bandage.. ....... 279
Tinea Kerion, by W. H. Fitch, M.D ...................... 478
Traumatic insanity, D. R. Brower, M.D......... .. ........ 609
Transfusion, case of, by F. W. Epley, M.D.................. 239
Tubercular meningitis, by C. W. Earle, M.D............... 11
Turnipseed, E. B., M.D., vesico-vaginial fistula, new instrument for.................... 65
Turnipseed, E. B., M.D., new instruments..... ............. 68
Tumors, multiple of the skin, accompanied by intense pruritus....................... 412
Tucker, J. I., M.D., Physicians' vacations....... .......... 299
Tucker, J. I., M.D., beef and ice cream........ . .......... 38
Tumor of Knee, recurrent...... 481
Typhlitic abscess, by Thos. F. Rochester, M.D.............. 73

U.

Ulcer of foot, obstinate clinical reports...................... 173
Urine, acute suppression of, with hiccough ............. 494
Uterine displacement, treatment of by stem pessaries......... 64
Uterus, pendent, clinical rep... 167
Uteri procidentia complete, case of, cure, by N. H. Church M.D ....................... 273
Uterus, double, with double conception ................ 556

PAGE.
Uterine fibroids, complications of, J. S. Everett, M.D. ........ 598

### V.

Vacations, physicians, by J. I. Tucker, M.D., .................. 297
Vaccino-syphilis; or the accidental transmission of syphilis by vaccine lymph........ 329
Varicose vessels, a simple method of obliteration of in rosacea ....................
Vertigo, laryngeal............ 213
Vesico-vaginal fistula, new instruments for.............. 65

PAGE.
Viola tricolor.................. 405
Vertiligo, incomplete.......... 387

### W.

Westphal, correction of Dr. Caldwell .................... 186
Woman's Med. College appointments ....................... 38

### Y.

Yellow-fever.................... 327
Yellow-fever, origin and spread of, N. S. Davis, M.D........ 574

### Books and Pamphlets Received.

Transactions of the Minnesota State Medical Society, 1872, 1874, 1875, 1877, 1879.

Medical Society of New Jersey. Transactions, 1879.

Consumption and How to Prevent It. By Thomas J. Mays, M.D. Cl., pp. 89. 1879. New York: G. P. Putnam & Sons.

Memorial Oration in Honor of Ephriam McDowell, the Father of Ovariotomy. By Samuel Gross, M.D., etc. Cl., pp. 77. 1879. Published by the Kentucky State Medical Society.

Transactions of the Indiana State Medical Society, 1879. Cl., pp. 252.

Yellow Fever: A Nautical Disease. Its Origin and Prevention. By John Gamgee. Cl., pp. 207. 1879. New York: D. Appleton & Co. Chicago: Jansen, McClurg & Co.

Diseases of Women. By Lawson Tait F. R. C. S. Second edition. Thoroughly revised and enlarged. Specially prepared for Wood's Library. Cl., pp. 192. 1879. New York: Wm. Wood & Co. Chicago: W. T. Keener.

The Treatment of Diseases by the Hypodermic Method: A Manual of Hypodermic Medication. By Roberts Bartholomew, M.A.. M.D., &c. Third edition. Enlarged. Cl., pp. 249. 1879. Philadelphia: J. B. Lippincott & Co. Chicago: Jansen, McClurg & Co.

Clinical Lectures on Diseases of the Urinary Organs, delivered at University College Hospital. By Sir Henry Thompson. Fifth edition. Cl., pp. 355. 1879. Philadelphia: Lindsay & Blakiston. Chicago: Jansen, McClurg & Co.

The Pathology and Treatment of Venereal Diseases. By Freeman J. Bumstead, M.D., LL.D, Fourth edition. Revised and enlarged by the author and Robert W. Taylor, A.M., M.D. Leather, pp. 835. 1879. Philadelphia: H. C. Lea. Chicago: Jansen, McClurg & Co.

System of Midwifery, including the Diseases of Pregnancy and the Puerperal State. By William Lersham, M. D. Third American edition, with additions by J. S. Parry, M.D. Leather, pp. 732. 1879. Philadelphia: H. C. Lea. Chicago: Jansen, McClurg & Co.

First Lines of Therapeutics: As Based on the Modes and Processes of Healing, as Occurring Spontaneously in Disease, and on the Modes and Processes of Dying, as Resulting Naturally from Diseases. By Alexander Harvey, M.A., M.D. Cl., pp. 278. 1879. New York: D. Appleton & Co. Chicago: Jansen, McClurg & Co.

A Ministry of Health, and Other Addresses. By B. W. Richardson, M.D., &c. Cl., pp. 354. 1879. New York: D. Appleton & Co. Chicago: Jansen, McClurg & Co.

Health Primer: The Skin and Its Troubles. Cl., pp. 94. 1879. New York: D. Appleton & Co. Chicago: Jansen, McClurg & Co.

Experimentallei Nachweis: Siner Freien Communication der Endolymphatischen und Perilymphatischen Räume des Menschlichen Orlabyrinthes, mit Extra Calynuthischen Intracramellen Räumen. By Dr. Weber Liel. 1879.

Atlas of Histology. By E. Klein, M.D., F.R.S., and E. Noble Smith, LL.D., C.P., &c. Part VII. Philadelphia: J. B. Lippincott & Co. Chicago: Jansen, McClurg & Co.

Early Medical Chicago: An Historical Sketch of the First Practitioners of Medicine, with the Present Faculties and Graduates Since their Organization, of the Medical Colleges of Chicago. By James N. Hyde, A.M., M.D.

Fourteenth Annual Report of the Chicago Hospital for Women and Children, for year ending March, 1879.

Tobacco Poisoning, and Its Effects on the Eyesight. By A. W. Calhoun, M.D. Published from the Transactions of the Medical Association of Georgia.

The Extirpation of the Ovaries for Some of the Disorders of Menstrual Life. By W. Goodell, A.M., M.D. Reprint from the transactions of the Medical Society of the State of Pennsylvania. 1879.

(Die Pochenkrankheit, Heilbar.) Ueber Masmatische Anstecke—Ung Mit Specialler Bezeihung Auf die Entstehung, und das Wesen der Pochenkrankheit. Nebest Angelie Eines Specilisch en Heilverfahrens Gegen de Pocknen von Dr. W. Hübner.

Report of the Resident Physician of Bingham Hall, a Hospital for the Insane.

Proceedings of the Association of Medical Officers of American Institution for Idiotic and Feeble-minded Persons, sessions 1878–1879.

Medical Legislation, being the Annual Address before the State Medical Society. By Nicholas Senn, M.D., President. Reprint from the transactions of the Wisconsin State Medical Society.

Minutes of the Medical Society of the County of New York, 1806–1878. Edited by A. E. M. Purdy, M.D. Part VI.

Lunacy Reforms: Historical Considerations. By E. C. Seguin, M.D. Reprint from Archives of Medicine, October, 1879.

Two Cases of Clonic Blepharospasms as Traumatic Reflex Neurosis. By F. C. Hotz, M.D. Reprint from *Amer. Jour. of Med. Sciences*, October, 1879.

Transactions of the Massachusetts Medico-Legal Society. Vol. I. No. 2. 1879.

Sylvester S. Bliss' Illustrated Catalogue of Surgical Instruments and Physicians' Goods.

An Examination of the Usual Signs of Dislocation of the Hip; also An Inquiry into the Proper Mode of Procedure when Dislocation of the Hip is Accompanied with Fracture of the Femur. By Oscar H. Allis, M.D. Reprint from the transactions of the Medical Society of the State of Pennsylvania for 1879.

---

WE have to ask the indulgence of our friends for the delay of this issue, due in part to the unusual number of cuts, called for at a late moment, and in part to changes in the typographical department. We expect to issue the January number of this journal, on the first prox.

### ANNOUNCEMENTS FOR THE MONTH.

### SOCIETY MEETINGS.

Chicago Medical Society—Mondays, Dec. 1 and 15.

West Chicago Medical Society—Mondays, Dec. 8 and 22.

### CLINICS.

MONDAY.

Eye and Ear Infirmary—2 p. m., Ophthalmological, by Prof. Holmes; 3 p. m., Otological, by Prof. Jones.

Mercy Hospital—2 p. m., Surgical, by Prof. Andrews.

Rush Medical College—2 p. m., Dermatological and Venereal, by Prof. Hyde; 3 p. m., Medical, by Dr. Bridge.

Woman's Medical College—3 p. m., Diseases of the Chest, Prof. Ingals.

TUESDAY.

Cook County Hospital—2 to 4 p. m., Medical and Surgical Clinics.

Mercy Hospital—2 p. m., Medical, by Prof. Hollister.

WEDNESDAY.

Chicago Medical College—2 p. m., Eye and Ear, by Prof. Jones.

Rush Medical College—3:30 to 4:30 p. m., Diseases of the Chest, by Dr. E. Fletcher Ingals.

THURSDAY.

Chicago Medical College—2 p. m., Gynæcological, by Prof. Jenks.

Rush Medical College—3 p. m., Diseases of the Nervous System, by Prof. Lyman.

Eye and Ear Infirmary—2 p. m., Ophthalmological, by Dr. Hotz.

Woman's Medical College—3 p. m., Surgical, by Prof. Owens.

FRIDAY.

Cook County Hospital—2 to 4 p. m., Medical and Surgical Clinics.

Mercy Hospital—2 p. m., Medical, by Prof. Davis.

SATURDAY.

Rush Medical College—2 p. m., Surgical, by Prof. Gunn.

Chicago Medical College—2 p. m., Surgical, by Prof. Isham; 3 p. m., Neurological, by Prof. Jewell.

Woman's Medical College—11 a. m., Ophthalmological, by Prof. Montgomery; 2 p. m., Gynæcological, by Prof. Fitch.

Daily Clinics, from 2 to 4 p. m., at the Central Free Dispensary, and at the South Side Dispensary.

